# Z-ligustilide preferentially caused mitochondrial dysfunction in AML HL-60 cells by activating nuclear receptors NUR77 and NOR1

**DOI:** 10.1186/s13020-023-00808-7

**Published:** 2023-09-21

**Authors:** Gen Liu, Zhi-gang Chen, Li-rong Yang, Yu-xia Rong, Qin Wang, Li Li, Qian-wei Lu, Ming-dong Jiang, Hong-yi Qi

**Affiliations:** 1 College of Pharmaceutical Sciences, College of Chinese Medicine, Southwest University, 2 Tiansheng Road, Beibei District, Chongqing, 400715 China; 2 Radiotherapy Department, Chongqing Ninth People’s Hospital, Chongqing, China

**Keywords:** Z-ligustilide, Acute myeloid leukemia, NUR77, NOR1, Mitochondrial dysfunction

## Abstract

**Background:**

Nuclear receptors NUR77 and NOR1 were identified as critical targets in acute myeloid leukemia (AML) therapy. Previously, we showed that Z-ligustilide (Z-LIG) selectively targeted AML by restoring NUR77 and NOR1. However, its downstream mechanisms are yet to be elucidated.

**Methods:**

SRB staining assay was used to measure cell viability. Cell apoptosis, mitochondrial membrane potential and mitochondrial reactive oxygen species were analyzed using flow cytometry. The potential targets of Z-LIG in AML HL-60 cells were evaluated by RNA sequencing. Changes in RNA levels were measured using quantitative RT-qPCR and western blot analysis was used to detect the expression of proteins.

**Results:**

Z-LIG preferentially induced mitochondrial dysfunction in HL-60 cells compared with 293T cells. Furthermore, RNA sequencing revealed that mitochondrial transcription and translation might be potential Z-LIG targets inhibiting HL-60 cells. NUR77/NOR1 overexpression significantly reduced the mitochondrial ATP and mitochondrial membrane potential and increased mitochondrial reactive oxygen species in HL-60 cells but not in 293T cells. Moreover, Z-LIG induced mitochondrial dysfunction by restoring NUR77 and NOR1 in HL-60 cells. Compared with HL-60 cells, the apoptosis-inducing activities of NUR77/NOR1 and Z-LIG were significantly reduced in HL-60 ρ0 cells depleted in mitochondrial DNA (mt-DNA). Moreover, NUR77/NOR1 and Z-LIG downregulated mitochondrial transcription and translation related proteins in HL-60 cells. Notably, Z-LIG remarkably reduced mitochondrial ATP in primary AML cells and showed anti-AML activity in mouse models of human AML.

**Conclusions:**

Collectively, our findings suggested that Z-LIG selectively induces mitochondrial dysfunction in AML HL-60 cells by restoring NUR77 and NOR1, a process associated with interference in mtDNA transcription.

**Supplementary Information:**

The online version contains supplementary material available at 10.1186/s13020-023-00808-7.

## Introduction

Acute myeloid leukemia (AML) is one of the most common types of leukemia and is most often encountered in patients > 65 years [1]. In 2023, 20,380 new AML cases are expected and 11,310 people will die from the disease [2]. Targeting metabolic differences between tumor and normal cells is an emerging anticancer therapeutic strategy [3]. AML cells possess more unique mitochondrial characteristics than normal hematopoietic progenitor cells and are highly dependent on mitochondrial oxidative phosphorylation (OXPHOS) [4], which requires mitochondrial respiratory chain subunits encoded by mitochondrial DNA (mtDNA) [5]. Hence, interfering with the synthesis of subunits leads to mitochondrial dysfunction and results in the apoptosis of AML cells.

NUR77 and NOR1 are two highly homologous orphan nuclear receptors encoded by the genes *NR4A1* and *NR4A3*. However, the roles of NUR77 and NOR1 in tumor progression are unclear [6]. NUR77 and NOR1 are critical tumor suppressors in myeloid malignancies. Reduced NUR77 expression has been detected frequently in patients with AML [7]. Moreover, NUR77 and NOR1 deletion in mice led to rapidly lethal AML [8]. Conversely, NUR77 upregulation has been found in various cancer cells and tissue samples, such as high-fat associated aortic endothelial cells, related to mitochondrial dysfunction, ATP energy production disorder, and apoptosis in these cells and tissues [9]. However, the roles of NUR77 and NOR1 in mitochondrial function and ATP synthesis in AML cells require further research.

Z-ligustilide (Z-LIG), the main component in *Rhizoma* Chuanxiong, exhibits various pharmacological activities [10]. Our previous research showed that Z-LIG could induce cell apoptosis by activating the NUR77/NOR1-BCL2 apoptotic mechanism [11]. Thus, we aimed to further investigate the effects of NUR77/NOR1 and Z-LIG on mitochondrial respiratory chain function and ATP synthesis in AML cells.

In this study, we explored the possible role of NUR77/NOR1-mediated mitochondrial dysfunction against AML and its correlation with the selective anti-AML activity of Z-LIG.

## Materials and methods

### Materials

Z-LIG (purity ≥ 98%, product number: PS010302, CAS number: 81944-09-4) was purchased from Push Bio-technology (Chengdu, China). The Apoptosis Detection Kit was obtained from Elabscience (Wuhan, China). The Intracellular ATP Assay Kit and JC-1 were purchased from Beyotime (Shanghai, China). MitoSOX™ Red was obtained from Invitrogen (Carlsbad, CA, USA). Details of the antibodies used are listed in Additional file 1: Table S1. The primer sequences are summarized in Additional file 2 (Tables S2) and Additional file 3 (Table S3). Unless otherwise specified, all other chemicals were purchased from Sigma-Aldrich (St, Louis, MO, USA).

### Cell culture

The AML cell line HL-60 (AML-M2) cells and human embryonic kidney cell line 293T were purchased from the Cell Bank of Chinese Academy of Science (Shanghai, China). Primary AML cells were isolated using Ficoll density centrifugation from recently diagnosed patients without prior therapy after obtaining informed consent. In this study, the collection and use of human tissue were approved by the Review Ethics Board of Chongqing Ninth People’s Hospital (Chongqing, China). Cells were grown in RPMI-1640, DMEM, or IMDM medium supplemented with 10% FBS and 1% penicillin/streptomycin in a 5% CO_2_ humidified incubator at 37 °C.

### Cell viability assay

After treatment, the cells were fixed with TCA (80% for suspension cells and 30% for adherent cells). Afterward, cells were stained with 0.4% sulforhodamine B (SRB) for 30 min. Then, SRB solutions were discarded, and tris base (10 mM) was added to dissolve the protein-bound dye. The absorbance at 490 nm was measured using a Synergy H1 plate reader (BioTek, USA).

### Cell apoptosis assay

Cells were harvested, washed in PBS, and resuspended in 1 × binding buffer. Then, cells were stained with Annexin V-FITC and propidium iodide for 20 min and measured using a flow cytometer (BD Biosciences, San Jose, CA, USA).

### Determination of mitochondrial membrane potential (MMP)

MMP was measured using the lipophilic JC-1 dye. Briefly, the cells were stained with JC-1 dye at 37 °C for 30 min and rinsed twice with 1 × incubation buffer. Subsequently, MMP was measured using flow cytometry.

### Determination of mitochondrial reactive oxygen species (ROS)

Mitochondrial ROS was detected using MitoSOX. Briefly, the cells were stained with MitoSOX(5 µM) in HBSS buffer at 37 °C for 30 min and rinsed twice with HBSS buffer. Subsequently, mitochondrial ROS was measured using flow cytometry.

### Measurement of mitochondrial ATP

Mitochondrial ATP was measured using the ATP Assay Kit as per manufacturer’s instructions. The cells were harvested and resuspended in lysis buffer according to the protocol. Fluorescein was added to the lysate, followed by firefly luciferase. Luminescence was measured using the Synergy H1 plate reader (BioTek, USA).

### RNA sequencing (RNA-seq)

The cDNA library was constructed and sequenced by Biomarker Technologies (Beijing, China) using the Illumina HiSeq 2500 platform. StringTie was used to calculate the gene expression level of each sample. DEGs were defined as fold change in expression |log2 (FC)| > 2.0 and Q ≤ 0.001.

### Transient transfection with siRNA and plasmids

Plasmids containing ov-NUR77, ov-NOR1, and an empty vector were obtained from Sino Biological (Beijing, China). The siRNA was obtained from Sigma-Aldrich. Transient transfection was performed using lipofectamine 2000 in an antibiotic-free medium. After 6 h, the cells were cultured in fresh medium and further experiments were performed.

### Western blotting

Total protein was isolated using 1 × RIPA lysis buffer supplemented with a protease inhibitors cocktail. Mitochondrial proteins were isolated with a commercial kit (Beyotime, China). Equal amounts of proteins dissolved in 1 × SDS-PAGE sample loading buffer were loaded per lane, resolved on an 8–15% SDS-PAGE gel, and transferred to PVDF membranes. The membranes were probed with primary antibodies overnight at 4 °C. After incubation, the membranes were washed in TBST and probed with secondary antibodies for 2 h at room temperature. Finally, the blots were detected using an enhanced chemiluminescence reagent (GE Healthcare, Sweden) according to the manufacturer’s specifications.

### Quantitative real-time PCR (qPCR) assay

Total RNA was isolated using TRIzol reagent (Beyotime, China), and cDNA was synthesized using PrimeScript RT Reagent Kit (TaKaRa, Japan). Subsequently, qPCR was performed using SYBR Green Master Mix (ThermoFisher, USA) and CFX96 Real-Time System (Bio-Rad, USA). The results were analyzed using the 2-ΔΔCT method.

### Generation of the HL-60 ρ0 cell line

The HL-60 ρ0 cell line (mtDNA-depleted cells) was generated as described previously [12]. Briefly, the HL-60 ρ0 cell line was derived from its parental cell line HL-60. The cells were selected using ethidium bromide (0.1 µg/mL) supplemented with sodium pyruvate (1 mM) and uridine (1 mg/mL) for 30 d. Lack of mtDNA was verified using PCR, and thereafter the addition of ethidium bromide was stopped. During drug treatment, pyruvate and uridine supplementation was discontinued.

### DNA extraction and PCR assay

The cells were harvested, DNA was isolated and mtDNA was amplified using PCR. The 420 bp amplified fragment was analyzed using electrophoresis on 1% agarose. The bands were visualized under a Gel Imaging System (Tanon, China).

### Animal model

This animal experiment was approved and supervised by the Institutional Animal Care and Use Committee of Southwestern University. Female NOD/SCID mice (5-week-old and 18–21 g body weight) were obtained from Hunan SJA Laboratory Animal Co. Ltd (Hunan, China). HL-60 cells in Matrigel were inoculated subcutaneously into mice (1 × 10^7^ cells/mice, n = 6 per group). When the tumor reached a volume of 50–100 mm^3^, the mice were randomized into two treatment groups: Z-LIG or vehicle was injected intraperitoneally once every other day for 2 weeks. Tumors were measured once every other day, and tumor volume was calculated as follows: (length × width^2^) × 0.5. When the tumor length reached 15 mm or 21 d after cell inoculation, the mice were euthanized, and the tumor mass was measured.

### Statistical analysis

All data were presented as the mean ± SD. Significant differences were examined using student’s t test or one-way ANOVA. A value of *P* < 0.05 was considered statistically significant. Prism 5.03 (GraphPad Software Inc., San Diego, CA, USA) was used to perform the calculations. Flow Jo 7.6 (Tree Star, Inc., Ashland, OR, USA) and ImageJ 1.8.0 (NIH, Bethesda, MD, USA) were used for image analysis.

## Results

### Z-LIG induced mitochondrial dysfunction in HL-60 cells

**Fig. 1:**
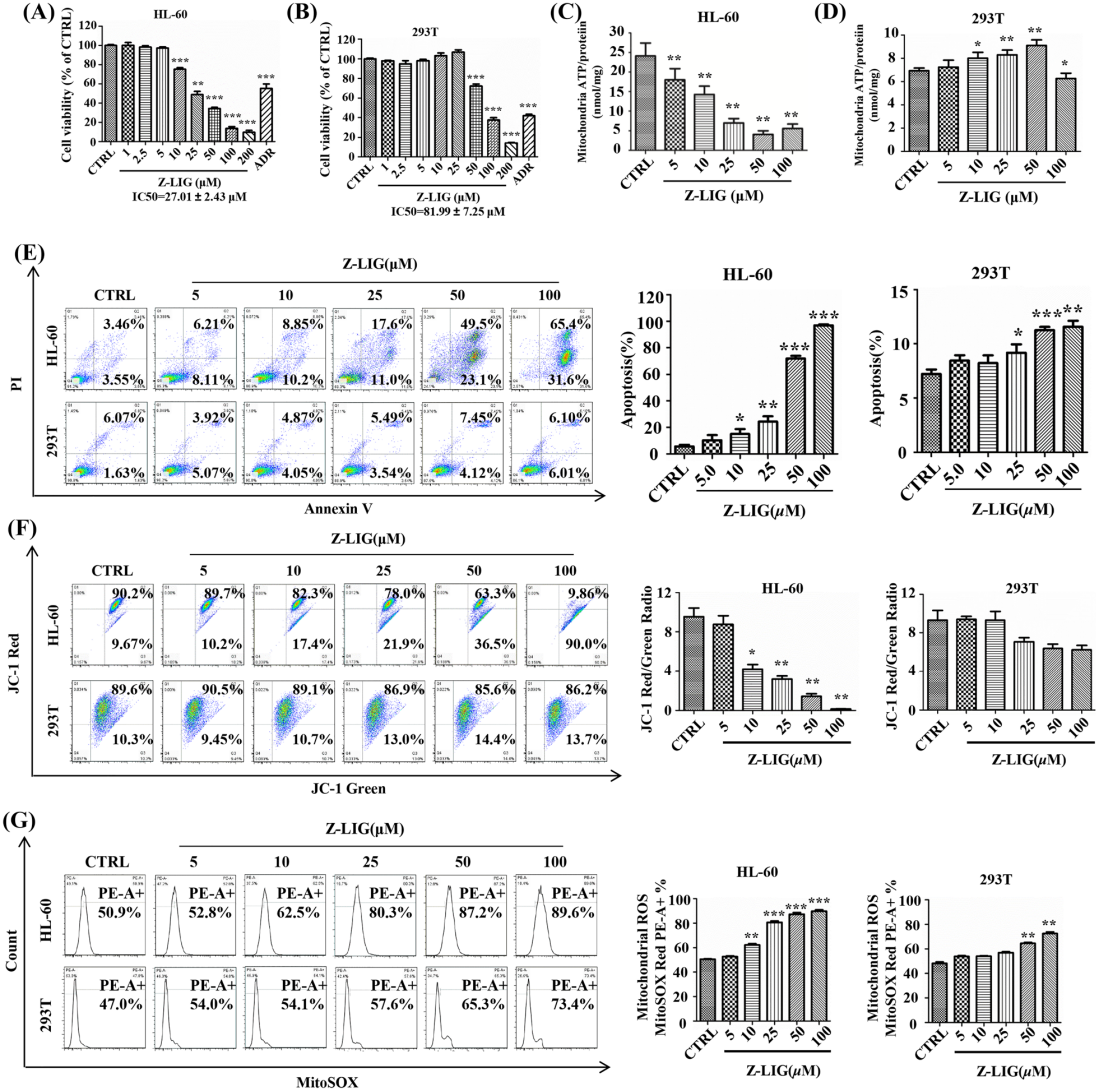
Z-LIG selectivity induced mitochondrial dysfunction in HL-60 cells. **A**, **B** Cell viability was measured using SRB after adriamycin (ADR) treatment (32 nM for HL-60 cells; 2.5 µM for 293T cells) or Z-LIG treatment for 72 h. **C**, **D** Cells were pretreated with 2-DG (5 mM) and sodium pyruvate (2 mM) for 1 h and stimulated with Z-LIG for 24 h; then, mitochondrial ATP was detected using luciferase assay. **E** Apoptosis was quantified using flow cytometry after Z-LIG treatment (48 h). **F**, **G** MMP and mitochondrial ROS induced by Z-LIG (24 h) were measured using flow cytometry after staining with the appropriate indicator. Values are presented as the means ± SD (n = 3 per group). *P < 0.05, ** *P* < 0.01, and *** *P* < 0.001 vs. CTRL

Z-LIG potently inhibited HL-60 (IC50 = 27.01 µM) cells (Fig. 1A) while relatively weak activities were observed in 293T (IC50 = 81.99 µM) cells (Fig. 1B). 293T cells, whereas show low ROS levels, have been used as control normal human cells in many studies on leukemia [13–16]. Furthermore, Z-LIG induced apoptosis in HL-60 cells in a concentration-dependent manner, with 97% apoptosis at 100 µM whereas that of 293T cells was < 12.11% (Fig. 1E). To validate whether Z-LIG influences mitochondrial function, we examined the mitochondrial ATP, MMP, and mitochondrial ROS levels after Z-LIG treatment. The results demonstrated that Z-LIG decreased the mitochondrial ATP levels in a concentration-dependent manner in HL-60 cells (Fig. 1C). A decrease of 83.12% was observed when treated with 50 µM of Z-LIG. However, the mitochondrial ATP production of 293T cells increased after Z-LIG treatment (10, 25, 50 µM), and decreased only by 12.6% after Z-LIG treatment (100 µM; Fig. 1D). Meanwhile, the red to green fluorescence ratio plummeted in HL-60 cells, which indicated that Z-LIG inhibited MMP (Fig. 1F). Conversely, this effect was not observed in 293T cells (Fig. 1F). Mitochondrial ROS levels increased in a concentration-dependent manner with Z-LIG treatment (10, 25, 50, 100 µM) in HL-60 cells (increased by 11.6%, 29.4%, 36.3%, and 38.7%, respectively; Fig. 1G). However, in 293T cells, there was no significant change in mitochondrial ROS levels after Z-LIG treatment (5–25 µM) but they increased by 18.3% and 26.4% at higher Z-LIG concentrations of 50 and 100 µM, respectively.

### RNA-seq identified mitochondrial function as Z-LIG target in HL-60 cells

**Fig. 2:**
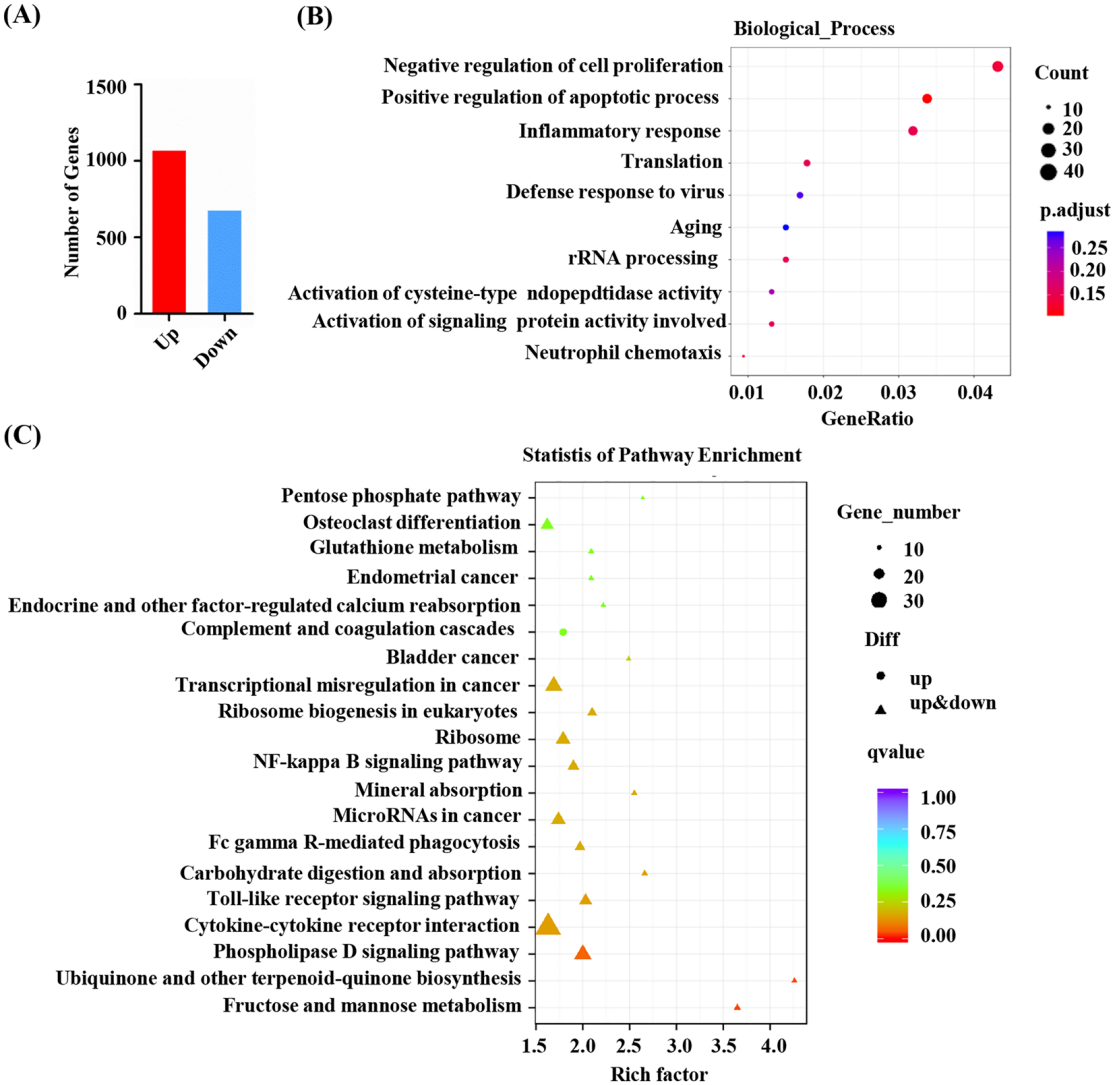
Potential target genes of Z-LIG based on RNA-seq. RNA-seq was performed in HL-60 cells after the treatment with vehicle or Z-LIG (25 µM for 24 h). **A** Statistical diagram of the number of DEGs. **B** GO enrichment analysis of DEGs. **C** KEGG pathway enrichment analysis of DEGs

We performed RNA-seq analysis with mRNA from HL-60 cells after incubation with vehicle or Z-LIG for 24 h. Compared with the vehicle group, 1766 differential expressed genes (DEGs) were detected in the Z-LIG treatment group, of which 1078 were upregulated and 688 were downregulated (Fig. 2A and Additional file 7: Fig. S1). The Gene Ontology (GO) classification and enrichment indicated that these DEGs were related to biological processes, such as translation, inflammatory response, negative regulation of cell proliferation, and positive regulation of apoptotic process (Fig. 2B). In addition, in Kyoto Encyclopedia of Genes and Genomes (KEGG) pathway enrichment analysis revealed that DEGs were mainly related to ubiquinone and other terpenoid-quinone biosynthesis, fructose and mannose metabolism, carbohydrate digestion and absorption, and other pathways (Fig. 2C and Additional file 4: Table S4).

Mitochondrial respiration is crucial for of cell metabolism. Tumor cells may adapt to environmental changes via the conversion between glycolysis and OXPHOS [17]. Hexokinase is one of the key rate-limiting enzymes in glycolysis. Hexokinase type 1 and type 3 isozyme expression was upregulated after Z-LIG treatment (Additional file 5: Table S5). In addition, the levels of PCK2, ENO2, ACSS2, ALDH1B1, and FBP1 were upregulated (Additional file 5: Table S5), all of which are factors involved in the regulation of glycolytic pathways.

Among the DEGs after 24 h of Z-LIG treatment, those encoding mitochondria-related proteins were highly abundant (5.55% of target mRNAs; Additional file 6: Table S6). mRNAs encoding components of OXPHOS complex I, III, and V, including NADH dehydrogenase 1 alpha subcomplex assembly factors 2, 4, and 6, UQCC3, ATP1A3, and ATP5G1, were downregulated (Additional file 6: Table S6). Similarly, mRNAs encoding maturation of primary transcription, mitochondrial translation elongation factor (TSFM), mitochondrial ribosomal proteins, and GTP binding protein 3 (GTPBP3), were also downregulated (Additional file 6: Table S6). These pathways are inextricably linked with the mitochondrial energy metabolism.

### Z-LIG induced mitochondrial dysfunction in HL-60 cells by restoring NUR77 and NOR1

**Fig. 3:**
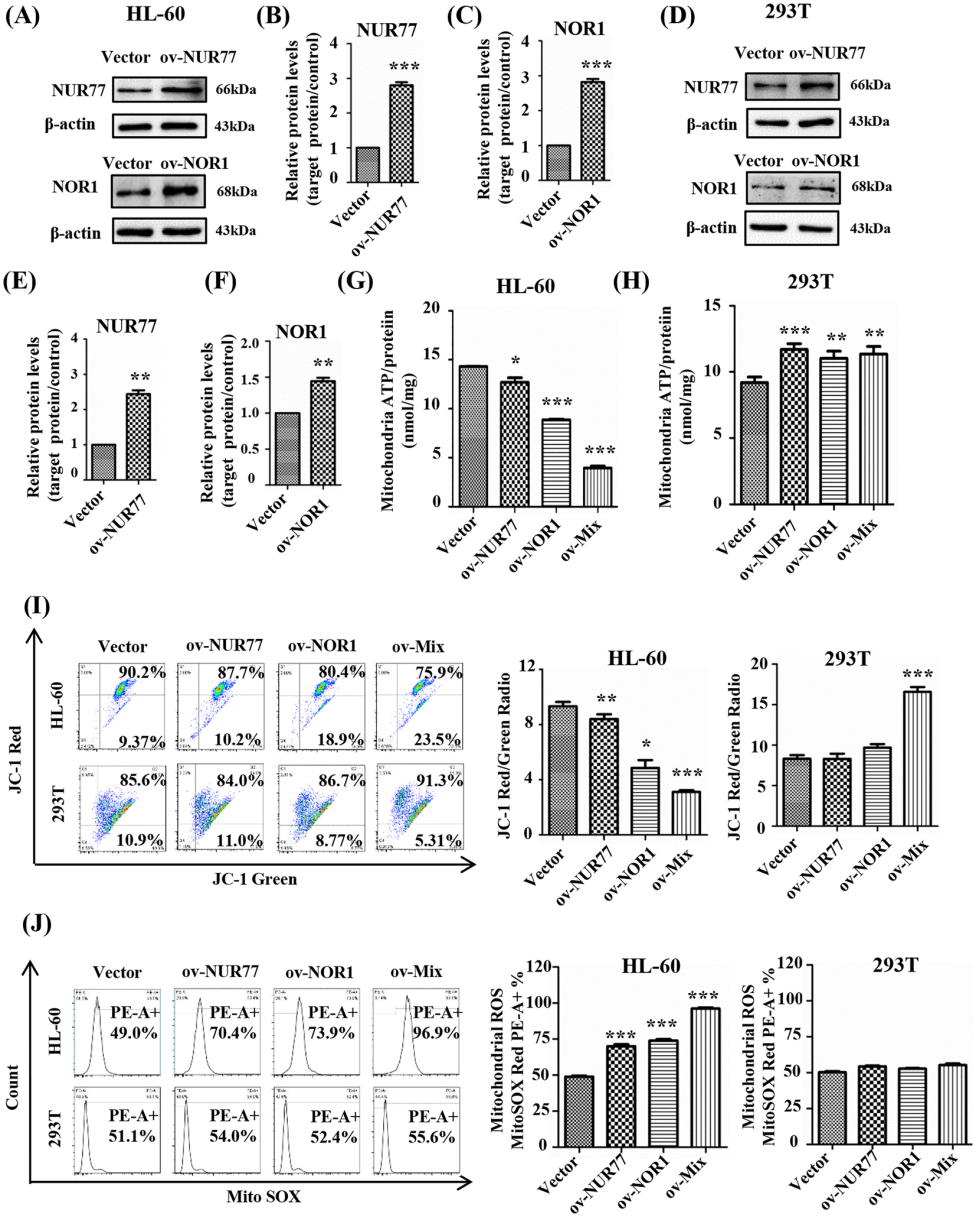
Effect of NUR77/NOR1 overexpression on cellular mitochondrial function. **A**–**F** Protein samples analyzed by western blotting for NUR77 and NOR1. **B** and **C** were densitometric analyses of the western blots shown in **A**. **E** and **F** were densitometric analyses of the western blots shown in **D**. **G**, **H** Cells were transfected with plasmid for 6 h and treated with 2-DG (5 mM) and sodium pyruvate (2 mM) for 24 h; then, mitochondrial ATP was determined using luciferase assay. **I**, **J** Cells were transfected with the indicated plasmid. MMP and mitochondrial ROS were measured after staining with the appropriate indicators. Values are presented as the means ± SD (n = 3 per group). *P < 0.05, ** *P* < 0.01, and *** *P* < 0.001 vs. Vector

We further determined the roles of NUR77 and NOR1 in Z-LIG-mediated mitochondrial dysfunction. NUR77 and NOR1 were overexpressed after treatment with the indicated plasmids (Fig. 3A–F). Both ov-NUR77 and ov-NOR1 decreased the mitochondrial ATP levels of HL-60 cells; the greatest inhibiting effect was observed by ov-Mix (co-transfection with ov-NUR77 and ov-NOR1; Fig. 3G). On the contrary, ov-NUR77, ov-NOR1, and ov-Mix significantly increased the mitochondrial ATP levels of 293T cells (*P* < 0.01; Fig. 3H). Moreover, ov-NUR77, ov-NOR1, and ov-Mix decreased the red to green fluorescence ratio of HL-60 cells from 9.63 to 8.60, 4.25, and 3.23, respectively, indicating a decrease in MMP (Fig. 3I). However, no significant effect on MMP was observed in 293T cells (Fig. 3I). In addition, compared with the control group, ov-NUR77, ov-NOR1, and ov-Mix increased mitochondrial ROS levels by 21.4%, 24.9%, and 47.9% in HL-60 cells, respectively (Fig. 3J). Notably, mitochondrial ROS levels increased by 2.9%, 1.3%, and 4.5% in 293T cells treated with ov-NUR77, ov-NOR1, and ov-Mix, respectively (Fig. 3J).

**Fig. 4:**
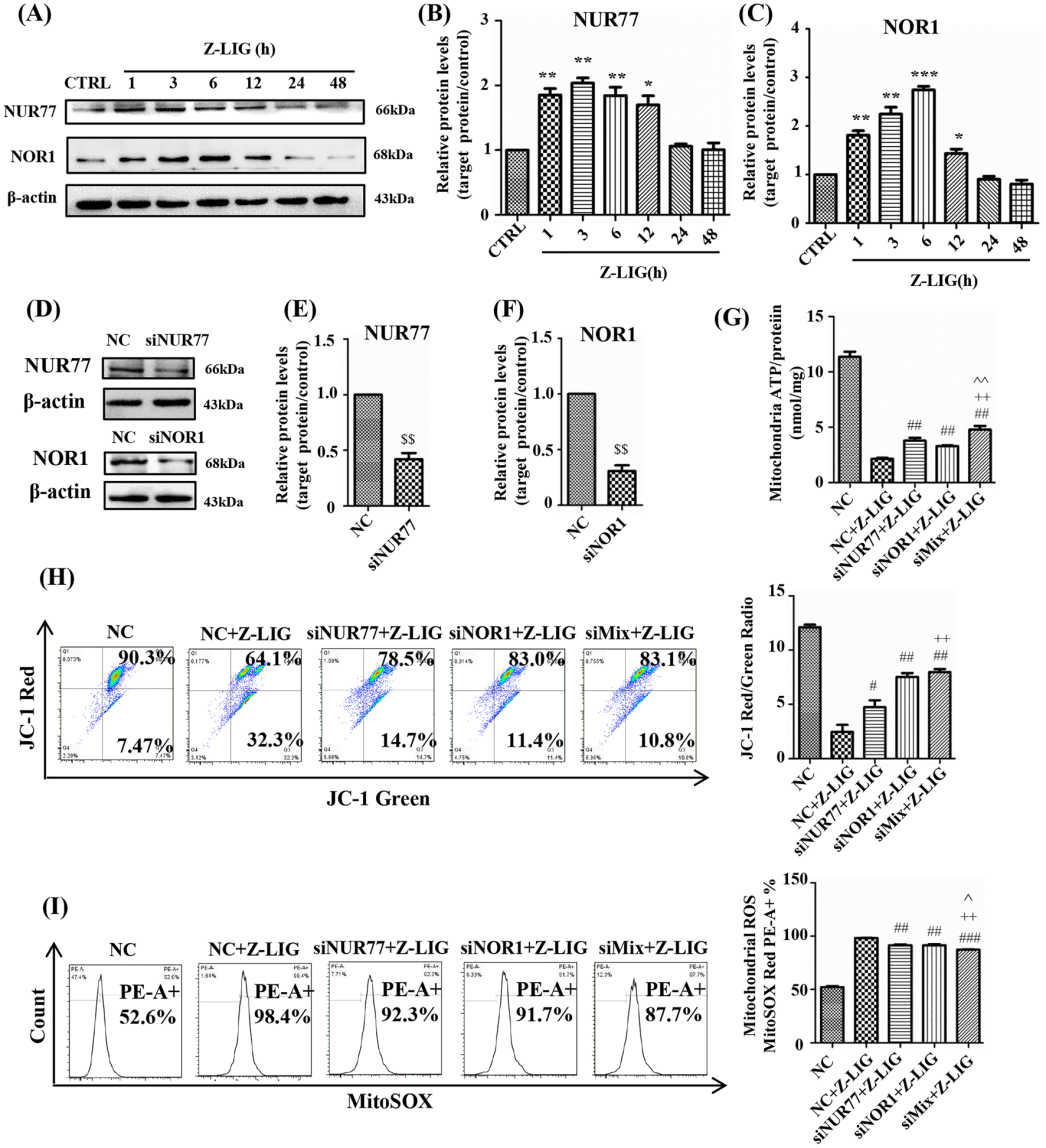
Effect of NUR77/NOR1 in Z-LIG-induced mitochondrial dysfunction. **A** Nur77 and Nor1 protein levels induced by Z-LIG (25 µM) at different time points analyzed with western blotting. **B**, **C** Densitometric analyses of the western blots in **A**. **D** HL-60 cells were transfected with siRNA for 24 h, and NUR77 and NOR1 levels were measured by western blotting. (E, F) Densitometric analyses of the western blots in **D**. **G** HL-60 cells were transfected with siRNA for 6 h. Thereafter, the cells were pretreated with 2-DG (5 mM) and sodium pyruvate (2 mM) for 1 h and stimulated with Z-LIG (25 µM) for 24 h. Mitochondrial ATP was detected using luciferase assay. **H**, **I** HL-60 cells were transfected with siRNA for 6 h and treated with Z-LIG (25 µM) for 24 h. MMP and mitochondrial ROS were detected after staining with the appropriate indicators. Values are presented as the means ± SD (n = 3 per group). *P < 0.05, ** *P* < 0.01, and *** *P* < 0.001 vs. CTRL. ^$$^*P* < 0.01 vs. NC. ^##^*P* < 0.01 vs. NC + Z-LIG. ^++^*P* < 0.01 vs. siNur77 + Z-LIG. ^^^^*P* < 0.01 vs. siNor1 + Z-LIG

Z-LIG rapidly increased NUR77 and NOR1 expression in HL-60 cells, and maximum expression was observed at 1–6 h (Fig. 4A–C). Then, siRNA was used to knockdown NUR77/NOR1 (Fig. 4D–F). siNUR77, siNOR1, and siMix (co-transfection with siNUR77 and siNOR1) partially reversed the Z-LIG-reduced mitochondrial ATP levels in HL-60 cells (Fig. 4G). Moreover, siNUR77, siNOR1 and siMix increased the red to green fluorescence ratio from 1.98 to 5.34, 7.28 and 7.69, respectively, thus indicating an increase in MMP (Fig. 4H). In addition, siNUR77 and siNOR1 caused a 6.1% and 6.7% reversion in Z-LIG-reduced mitochondrial ROS levels, respectively (Fig. 4I). Most importantly, siMix exhibited the best effect, with a 10.7% reversion (Fig. 4I).

### mtDNA was involved in Z-LIG and NUR77/NOR1 mediated HL-60 cells inhibition

**Fig. 5:**
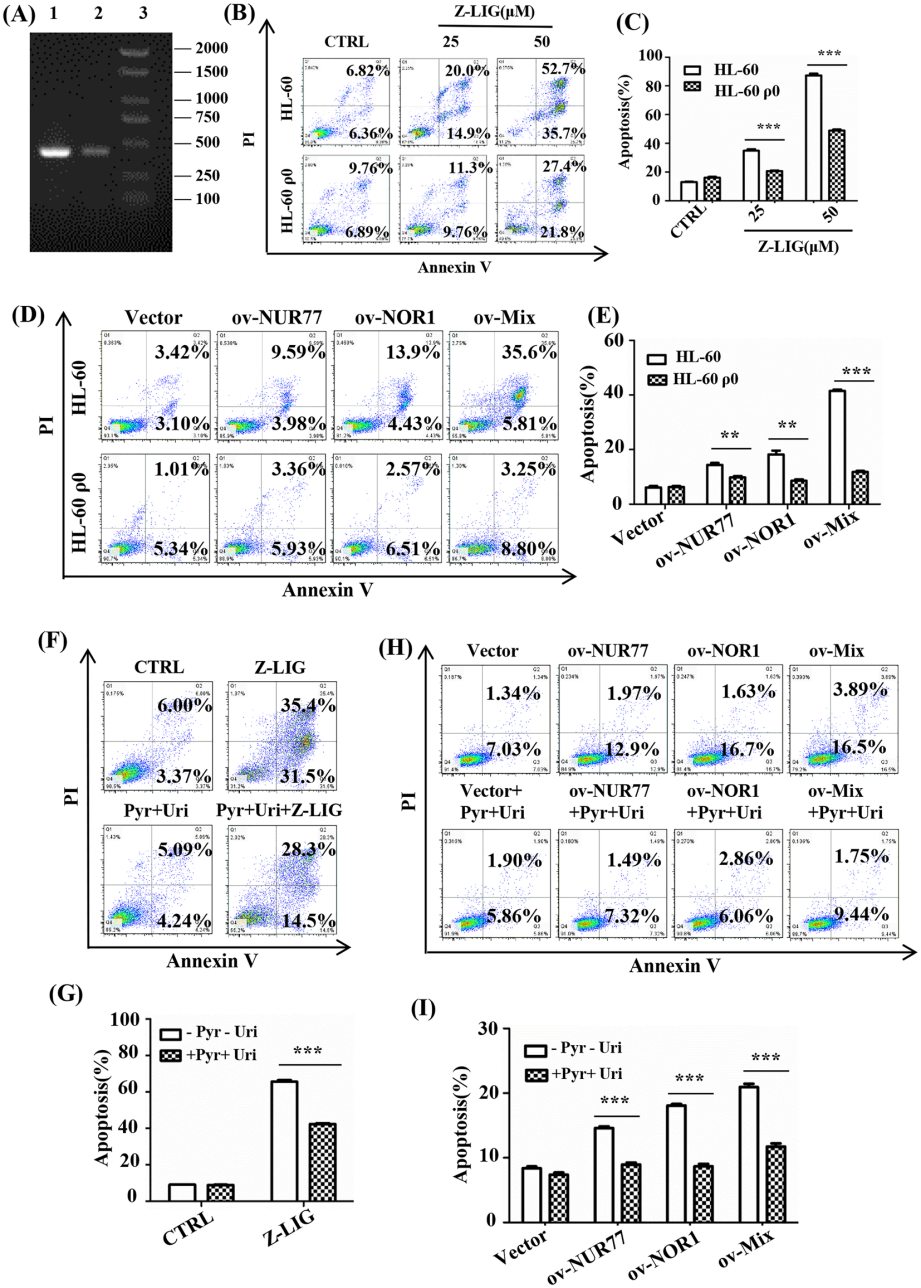
HL-60 ρ0 cells were resistant to NUR77/NOR1 overexpression or Z-LIG treatment. **A** The whole genome DNA of HL-60 and HL-60 ρ0 cells was isolated for PCR. (1) HL-60 cell expansion; (2) HL-60 ρ0 cell expansion; (3) Molecular standard. **B**, **E** Cells were treated with Z-LIG or transfected with the plasmid for 48 h; then, apoptosis was measured using flow cytometry. **F**–**I** HL-60 cells were pretreated with uridine (1 mg/mL) and pyruvate (1 mM) for 1 h and stimulated with Z-LIG (25 µM) or transfected with the plasmid for 48 h. Apoptosis was measured using flow cytometry. Values are presented as the means ± SD (n = 3 per group). ** *P* < 0.01 and *** *P* < 0.001 vs. indicated group

To validate whether the mitochondrial respiratory chain participates in the apoptosis of HL-60 cells induced by Z-LIG or NUR77/NOR1, mtDNA-depleted (ρ0) cells were generated by exposing the cells to ethidium bromide for 30 d (Fig. 5A). Compared to HL-60 cells, Z-LIG had a weaker effect on inducing apoptosis in mitochondrial respiratory chain-deficient HL-60 ρ0 cells (Fig. 5B, C). Similarly, compared with HL-60 cells, ov-NUR77, ov-NOR1, and ov-Mix had significantly reduced apoptosis-inducing activity in HL-60 ρ0 cells (Fig. 5D, E).

As previously reported, we attempted to rescue Z-LIG treated cells or NUR77/NOR1 overexpressing cells with uridine and pyruvate [12]. Adding these two supplements notably reversed the Z-LIG or NUR77/NOR1 overexpression induced apoptosis (Fig. 5F–I), indicating that Z-LIG or NUR77/NOR1 overexpression induced cell apoptosis in HL-60 cells by disturbing the mitochondrial electron transport chain.

**Fig. 6:**
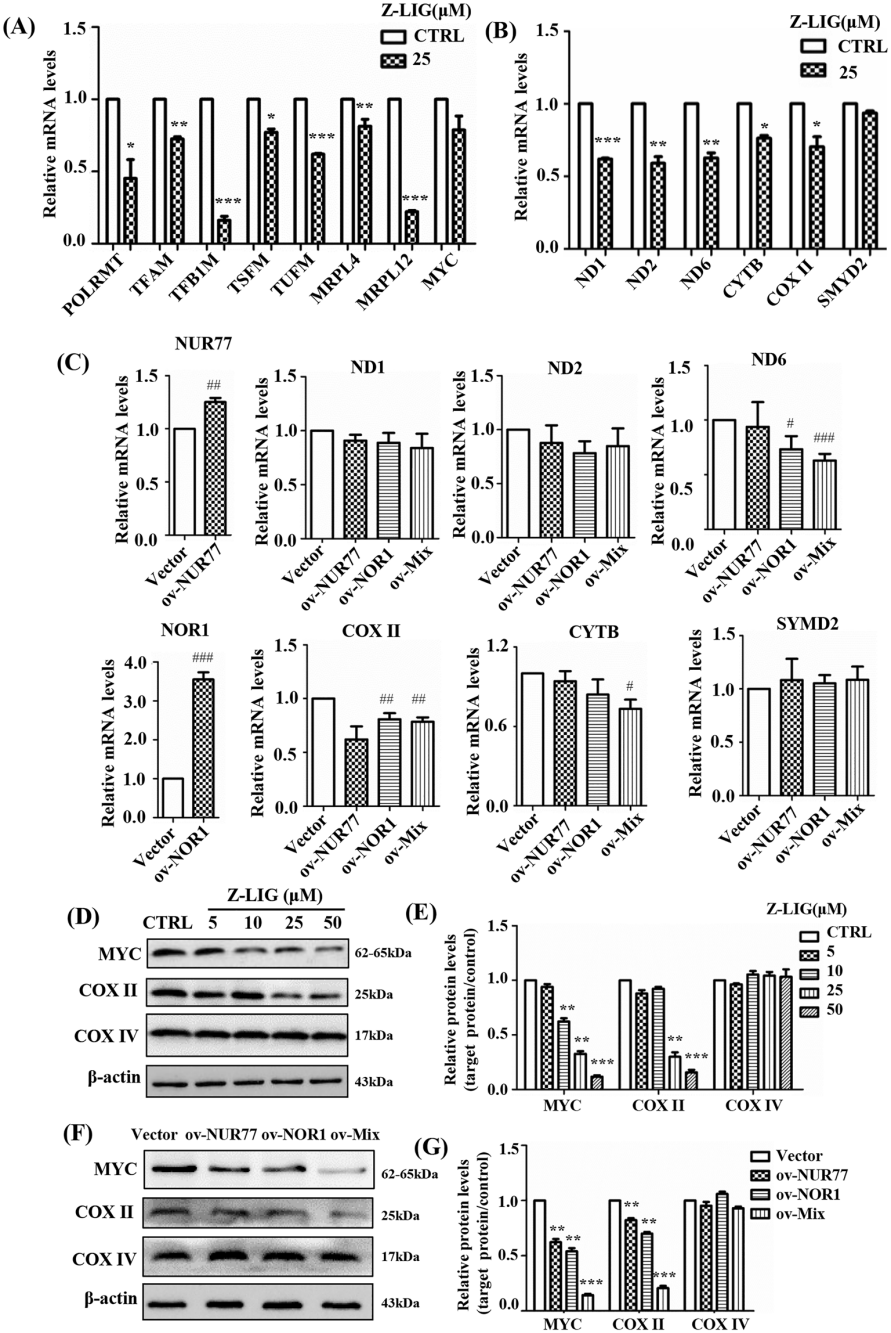
Effects of Z-LIG and NUR77/NOR1 on mitochondrial transcription and translation in HL-60 cells. **A**–**C** The mRNA levels were measured using qPCR after treatment with Z-LIG (24 h) or transfection with the plasmid in HL-60 cells. **D**–**G** HL-60 cells were transfected with the plasmid or treated with Z-LIG as indicated for 48 h. Total protein samples were analyzed using western blotting for MYC, COX II, and COX IV. E was densitometric analyses of the western blots shown in **D**, and **G** was densitometric analyses of the western blots shown in **F**. Values are presented as the means ± SD (n = 3 per group). *P < 0.05, ** *P* < 0.01, and *** *P* < 0.001 vs. CTRL. ^#^*P* < 0.05, ^##^*P* < 0.01, ^###^*P* < 0.001 vs. Vector

POLRMT, TFAM, TFB1M and TFB2M are related to mitochondrial transcription [18]. The mRNA levels of POLRMT, TFAM, and TFB1M decreased significantly in the Z-LIG group (Fig. 6A). In addition, the mRNA levels of TSFM, TUFM, MRPL4, and MRPL12, which are essential for mitochondrial translation [18], were significantly lower in the Z-LIG group than in the control group (*P* < 0.01; Fig. 6A). POLRMT is a critical downstream target of MYC [19]. Our findings showed that MYC mRNA levels were decreased as well (Fig. 6A). Moreover, the mRNA levels of ND1, ND2, ND6, CYTB, and COX II, which are subunits of the mitochondrial respiratory chain complex encoded by the mtDNA, decreased after Z-LIG treatment (Fig. 6B). However, the mRNA levels of SMYD2, encoded by nuclear DNA, did not decrease (Fig. 6B). NOR1 overexpression reduced ND6 and COX II transcription levels (Fig. 6C). Notably, ov-Mix had a better effect as it decreased the mRNA levels of ND6, COX II, and CYTB (Fig. 6C).

COX II and COX IV are subunits of respiratory complex IV; COX IV is encoded by the nuclear genome [20]. Z-LIG treatment caused a preferential decrease of COX II as compared to COX IV (Fig. 6D, E). Z-LIG significantly reduced MYC expression (Fig. 6D, E). Moreover, ov-NUR77, ov-NOR1, and ov-Mix downregulated MYC and COX II expression (*P* < 0.01), whereas no significant change was observed in COX IV (*P* > 0.05; Fig. 6F, G). Taken together, our findings supported that mtDNA was involved in the Z-LIG and NUR77/NOR1 mediated inhibition of AML cells.

### Z-LIG inhibited mitochondrial function in primary AML cells

**Fig. 7:**
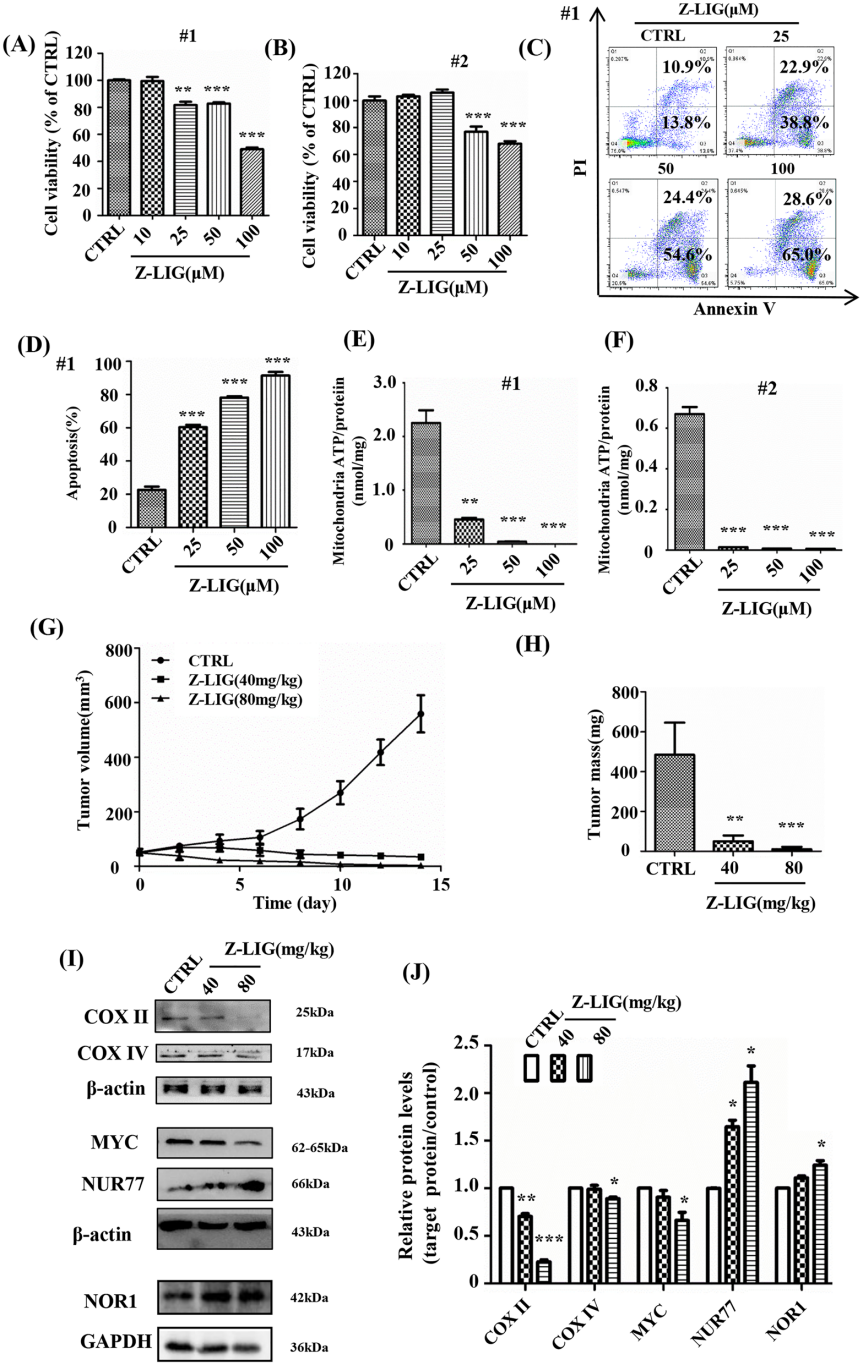
Anti-AML activity of Z-LIG in primary AML cells and NOD/SCID mice. **A**, **B** Cell viability of primary AML cells measured using SRB after treatment with Z-LIG (72 h). **C**, **D** The number of apoptotic cells was measured after treatment with Z-LIG (48 h). **E**, **F** Cells were pretreated with 2-DG (5 mM) and sodium pyruvate (2 mM) for 1 h, and after treatment with Z-LIG (25 µM for 24 h), mitochondrial ATP was detected using luciferase assay. **G**, **H** Tumor volume and mass (n = 6 per group). (I) COX II, COX IV, MYC, Nur77 and NOR1 levels in the tumor analyzed with western blotting. **J** Densitometric analyses of the western blots in H. Values are presented as the means ± SD (n = 3 per group). *P < 0.05, ** *P* < 0.01 and *** *P* < 0.001 vs. CTRL

Human primary AML cells were isolated to further confirm the effect of Z-LIG on mitochondrial function. Z-LIG (50–100 µM) inhibited cell viability in primary AML cells #1 and #2 (*P* < 0.001; Fig. 7A, B). Moreover, Z-LIG induced cell apoptosis in primary AML cells #1 (Fig. 7C, D). Notably, luciferase assay revealed that Z-LIG reduced the mitochondrial ATP production in primary AML cells #1 and #2 (*P* < 0.001; Fig. 7E–F).

### Z-LIG inhibited AML cells in xenograft models of human AML

The anti-AML activity of Z-LIG was further assessed in vivo. HL-60 cells were transplanted subcutaneously into NOD/SCID mice. After 7 days, when tumor volumes reached 50–100 mm^3^, the mice were treated with vehicle or Z-LIG. Compared with the control group, Z-LIG delayed tumor growth (*P* < 0.01; Fig. 7G, H). Moreover, there were no apparent changes in the organs at necropsy. After excising the tumors, protein was extracted to investigate changes in protein expression. Compared with the control group, Z-LIG treatment reduced the expression of mitochondrial-translated COX II but did not affect the expression of nuclear-translated COX IV (Fig. 7I, J). Besides, Z-LIG reduced the expression of MYC and increased the expression of NUR77 and NOR1 compared with the control group (Fig. 7I, J).

## Discussion

In our previous study, Z-LIG showed a strong inhibitory effect on cell growth in different AML cell lines and extremely weak cytotoxicity in other types of tumor cells [11]. This study demonstrated that Z-LIG exerted inhibitory effects in human AML HL-60 cells but not in normal 293T cells. In addition, Z-LIG (10–100 µM) selectively induced apoptosis in HL-60 cells but had little effects in normal cells. These results indicated that Z-LIG selectively inhibited AML HL-60 cells. Compared with normal hematopoietic progenitor cells, AML cells possess unique mitochondrial characteristics, such as higher levels of oxidative phosphorylation, larger mitochondrial mass, more copies of mtDNA and reduced spare reserve capacity of the mitochondrial respiratory chain [21, 22]. Thus, AML cells may require a higher energy production to maintain a high growth and proliferation rate. In this study, RNA-seq in HL-60 cells after Z-LIG treatment showed that 98 genes (5.55%) of the 1766 DEGs regulated by Z-LIG are related to mtDNA replication, transcription, and translation, almost all of which were inhibited. Importantly, it was reported that tigecycline inhibited AML cell as a mitochondrial translation inhibitor [23]. Therefore, we focused on the effects of Z-LIG on mitochondrial dysfunction and the potential mechanisms in this study.

NUR77 and NOR1 belong to the NR4A orphan nuclear receptor subfamily genes encoding proteins. Moreover, NR4A is an ancient and evolutionarily conserved subfamily of immediate early genes, and can be rapidly induced by environmental stimulus signals [24]. Different studies have shown that NUR77 and NOR1 were rapidly induced after 1–14 h of treatment [25–27]. In our previous study, we found that Z-LIG restored NUR77 expression by decreasing recruitment of HDAC1, HDAC4/5/7 and MTA1, and increasing p300 to Nur77 promoter [11]. And Z-LIG also reduced HDAC1 and HDAC3 and increases p-CREB to the NOR1 promoter recruitment, restored the expression of NOR1 [11]. NR4A receptors can alter the expressions of downstream genes associated with apoptosis, such as Fas, TRAIL, and MYC [27–29]. To determine the relationship between NUR77/NOR1 and mitochondrial function, we overexpressed or knocked down NUR77/NOR1 with Z-LIG treatment to determine its effect on mitochondrial ATP, MMP and mitochondrial ROS at 24 h. Our results showed that NUR77/NOR1 manipulation had a significant effect on downstream endpoint events, indicating that NUR77/NOR1 was closely associated with mitochondrial function and played a key role in Z-LIG-mediated mitochondrial dysfunction. Besides, Boudreaux et al. identified MYC as a direct downstream target of NR4A1 and NR4A3 [27].The expression of MYC is strictly regulated during normal cell proliferation, while abnormal regulation is often seen in tumor cells [30, 31]. It has been reported that MYC was highly expressed in the bone marrow of more than 90% AML patients, and the expression of MYC protein can be an important prognostic factor in AML patients with high risk of recurrence [32]. This may provide an explain for the result of our study that overexpression of NUR77 and NOR1 showed the different results in HL-60 cells and 293T cells.

Mitochondrial respiratory chain subunits synthesized via mitochondrial transcription and translation are essential for mitochondrial OXPHOS [33]. Cells mainly generate ATP via mitochondrial OXPHOS [34]. An electrochemical gradient generated by mitochondrial respiratory chain establishes MMP [35]. ROS is a byproduct of mitochondrial respiratory chain and generated when the chain is inefficient. Respiratory chain inhibitors have been reported to induce a rapid ROS generation and lead to cell death [36]. Loss of NUR77 and NOR1 expression is common in AML progression, and restoration of NUR77 and NOR1 may offer a meaningful therapy [7]. In addition, there is a close association between NUR77 and mitochondrial function. NUR77 was found to be highly expressed on brain tissue in a cerebral ischemia model [37], aortic endothelial cells in an atherosclerosis model [9], and hepatocytes in an animal model of nonalcoholic fatty liver [38], and directly associated with mitochondrial dysfunction, impaired ATP energy production, and apoptosis. The findings of the above study proved that NUR77 expression was negatively correlated with mitochondrial respiratory chain function and ATP synthesis in these disease models. In our study, Z-LIG and NUR77/NOR1 overexpression preferentially decreased the MMP and mitochondrial ATP levels while increasing mitochondrial ROS levels in HL-60 cells compared with 293T cells. In our another unpublished study, we found that N-acetyl-Lcysteine (NAC, a ROS scavenger) pretreatment partly rescued Z-LIG-induced cell viability inhibition and cell apoptosis in HL-60 cell. Moreover, in this study the Z-LIG-induced mitochondrial dysfunction was attenuated by NUR77/NOR1 knockdown in HL-60 cells. These results indicated that Z-LIG and NUR77/NOR1 overexpression may cause mitochondrial dysfunction in HL-60 cells. Moreover, the disruption of mitochondrial function caused by Z-LIG may be mediated by NUR77 and NOR1 activation in HL-60 cells. AML cells have a higher mitochondrial mass than normal hematopoietic cells, and the 13 mtDNA-encoded subunits of the electron transport chain are critical for OXPHOS [4]. Our work demonstrated that Z-LIG and NUR77/NOR1 overexpression had an impact on mtDNA transcription and translation. Previous studies demonstrated that NR4A tumor suppression in AML is closely related to MYC transcriptional repression [27]. Moreover, MYC protein expression is a key prognostic factor in untreated AML patients, especially those at a higher risk of relapse [32]. In this study, both Z-LIG and NUR77/NOR1 overexpression downregulated MYC protein expression. Eukaryotic cells remove damaged mtDNA, proteins, and lipids via balanced mitochondrial fusion and fission to maintain the normal morphology of mitochondria [39]. Disrupting this dynamic balance may lead to mitochondrial dysfunction and eventually apoptosis [40]. In Additional file 8: Fig. S2, Mdivi-1 (mitochondrial fission blocker) rescued the inhibition of cell viability caused by Z-LIG. Moreover, apoptosis induced by Z-LIG was reduced. MFN1, MFN2, and OPA1 play key roles in mitochondrial fusion, while MFF, FIS1, and DRP1 in mitochondrial fission [41]. In our results, Z-LIG decreased MFN1, MFN2, and OPA1 expression, increased MFF and FIS1 protein levels. Z-LIG promoted the phosphorylation of DRP1 at serine 616 position and the mitochondrial translocation of DRP1. These results suggested that Z-LIG induced mitochondrial division and affected the mitochondrial dynamic in HL-60 cells. The severe energy impairment resulting from the inactivation of mitochondrial ATP synthase is an early event in cell death [42, 43]. This was also demonstrated in the present study, where Z-LIG significantly reduced mitochondrial ATP levels in HL-60 cells at 5 µM and significantly inhibited cell survival at 10 µM. However, in primary AML cells, 50 µM of Z-LIG inhibited mitochondrial ATP production almost completely while inhibiting cell survival by only ~ 20%. This observation indicates that mitochondrial ATP levels are more sensitive to Z-LIG in primary AML cells than in HL-60 cells. However, the precise reason for this finding needs to be elucidated. Previous research demonstrated that tigecycline inhibited mitochondrial translation in AML cells, and that cells from different AML patients differ in their sensitivity [23]. More importantly, there were no significant differences in disease status or cytogenetic risk between groups with different sensitivities [23]. In this study, Z-LIG significantly reduced the mitochondrial ATP production in two primary AML cells. This result suggested that the effect of Z-LIG on mitochondrial function in AML cells requires future research. NOD/SCID mice are immunodeficient animals lacking T and B lymphocytes, facilitating the construction of leukemia models [44]. This study demonstrated that Z-LIG inhibited growth and mitochondrial function of HL-60 cells in vivo. Besides, our previous studies indicated that Z-LIG could effectively prolonged the survival of AML animal models [11].

## Conclusion

In summary, our study demonstrated that Z-LIG preferentially induced mitochondrial dysfunction in HL-60 cells rather than 293T cells. Furthermore, we established the presence of a strong correlation between mitochondrial dysfunction and overexpression of NUR77/NOR1 in HL-60 cells. Moreover, NUR77/NOR1-mediated mitochondrial dysfunction was critical for the anti-AML activity of Z-LIG.

Thus, Z-LIG effectively suppressed AML HL-60 cells by causing NUR77/NOR1-mediated mitochondrial dysfunction, and may potentially serve as a novel therapeutic agent for AML-M2.

### Supplementary Information

**Additional file 1: Table S1.** Antibody list.

**Additional file 2: Table S2.** RT-qPCR primer sequence list.

**Additional file 3: Table S3.** PCR primer sequence list.

**Adtional file 4: Table S4.** KEGG enrichment results of DEGs.

**Additional file 5: Table S5.** A subset of mRNAs regluating glycolysis that are translationally up-regulated following Z-LIG treatment.

**Additional file 6: Table S6.** A subset of mRNAs encoding mitochondrial proteins that are translationally suppressed after Z-LIG treatment.

**Additional file 7: Fig. S1.** Potential target genes of Z-LIG based on RNA-seq.

**Additional file 8: Fig. S2.** Z-LIG induced mitochondrial division in HL-60 cells.

## Data Availability

The data used to support the findings of this study are available from the corresponding author upon request.

## Abbreviations

2-DG: 2-Deoxy-d-glucose
ADR: Adriamycin
AML: Acute myeloid leukemia
DEGs: Differential expressed genes
MMP: Mitochondrial membrane potential
mtDNA: Mitochondrial DNA
NC: Non-specific control
OXPHOS: Oxidative phosphorylation
ROS: Reactive oxygen species
SRB: Sulforhodamine B
Z-LIG: Z-ligustilide
