# Supplementary material for: Z-ligustilide preferentially caused mitochondrial dysfunction in AML HL-60 cells by activating nuclear receptors NUR77 and NOR1
Source: Chin Med. 2023 Sep 21;18:123. doi: 10.1186/s13020-023-00808-7 (PMC10512564; doi:10.1186/s13020-023-00808-7)
Supplement: Supplementary file 1 — Additional file 1: Table S1. Antibody list. [file 13020_2023_808_MOESM1_ESM.doc]

**Additional file 1:**

**Table S1**

Antibody list

| **Antibody** | **Item number** | **Manufacturer** |
| --- | --- | --- |
| NUR77 | bs-3513R | Bioss |
| NOR1 | ab94507 | Abcam |
| MYC | 10828-1-AP | Proteintech Group, Inc |
| COX II | BS90888 | Bioworld Technology, Inc. |
| COX IV | 4850 | Cell Signaling Technology |
| OPA1 | sc-393296 | Santa Cruz Biotechnology |
| MFN1 | sc-166644 | Santa Cruz Biotechnology |
| MFN2 | sc-515647 | Santa Cruz Biotechnology |
| p-DRP1 Ser616 | bs-12702R | Bioss |
| DRP1 | sc-271583 | Santa Cruz Biotechnology |
| MFF | sc-398617 | Santa Cruz Biotechnology |
| FIS1 | sc-376447 | Santa Cruz Biotechnology |
| HSP60 | WL03647 | Wanlei Biotechnology |
| Anti-Mouse IgG | A4416 | Sigma-Aldrich |
| Anti-Rabbit IgG | SAB3700852-2MG | Sigma-Aldrich |
| OSCP1/NOR1 | 12598-1-AP | Proteintech Group, Inc |
| GAPDH | CL594-60004 | Proteintech Group, Inc |
| β-actin | TA-09 | Zhongshan Jinqiao Biotechnology |
