# Supplementary material for: Z-ligustilide preferentially caused mitochondrial dysfunction in AML HL-60 cells by activating nuclear receptors NUR77 and NOR1
Source: Chin Med. 2023 Sep 21;18:123. doi: 10.1186/s13020-023-00808-7 (PMC10512564; doi:10.1186/s13020-023-00808-7)
Supplement: Supplementary file 2 — Additional file 2: Table S2. RT-qPCR primer sequence list. [file 13020_2023_808_MOESM2_ESM.doc]

**Additional file 2:**

**Table S2**

RT-qPCR primer sequence list

| **Gene** | **Forward(5’-3’)** | **Reverse(5’-3’)** |
| --- | --- | --- |
| ND1 | CATTCCTAATGCTTACCGAACG | GTAGAGGGTGATGGTAGATGTG |
| ND2 | GGTTGCTTGCGTGAGGAAAT | AACCCTCGTTCCACAGAAGCT |
| ND6 | ATTTAGGGGGAATGATGGTTGT | CAATAGGATCCTCCCGAATCAA |
| CYTB | CGCATGATGAAACTTCGGCT | ATTTGGAGGATCAGGCAGGC |
| COX II | CGCATCCTTTACATAACAGACG | TAGGAGTTGAAGATTAGTCCGC |
| SMYD2 | TCATAGCTGTTGCCCCAATG | TCTGCCAGGGTCCCTTTGTA |
| MRPL4 | AAAGTCGAGCTCCCGGTA | AACCTGGTGCAGTATGTCC |
| TUFM | TACACTAGAGCGTGGCATTTTA | CTCTTGTGGAACATCTCAATGC |
| TFAM | TTCCAAGAAGCTAAGGGTGATT | AGAAGATCCTTTCGTCCAACTT |
| TSFM | ACTCAAGGATCAGTTGGCTTTA | GTGTTCTGCAAGAAGTTGGATT |
| MYC | CGACGAGACCTTCATCAAAAAC | CTTCTCTGAGACGAGCTTGG |
| POLRMT | GCAAGACCAAGACCGCAGGAAG | GCTACCATCTCCACTGCCACATTC |
| MRPL12 | AGGAGCAGCGGCCATCAGAG | AGAGTGAGGCTGGCGATGTCC |
| TFB1M | CGTAGTCGCCTCTCTGTTATGG | GCTGCTCTATCTTGGGCTGTATC |
| NUR77 | AGAGTTTGACACCTTCCTCTAC | GAAGTCCTCGAACTTGAAGGAG |
| NOR1 | CTACTCGAGCAACTACGAACTC | GACTGCTTGAAGTACATGGAGG |
| GAPDH | TGTTGCCATCAATGACCCCTT | CTCCACGACGTACTCAGCG |
