# Supplementary material for: Z-ligustilide preferentially caused mitochondrial dysfunction in AML HL-60 cells by activating nuclear receptors NUR77 and NOR1
Source: Chin Med. 2023 Sep 21;18:123. doi: 10.1186/s13020-023-00808-7 (PMC10512564; doi:10.1186/s13020-023-00808-7)
Supplement: Supplementary file 3 — Additional file 3: Table S3. PCR primer sequence list. [file 13020_2023_808_MOESM3_ESM.doc]

**Additional file 3:**

**Table S3**

PCR primer sequence list

| **Gene** | **Forward(5’-3’)** | **Reverse(5’-3’)** |
| --- | --- | --- |
| COX II | GGTCTATCACCCTATTAACCAC | CTGTTAAAAGTGCATACCGCCA |
