# Supplementary material for: Z-ligustilide preferentially caused mitochondrial dysfunction in AML HL-60 cells by activating nuclear receptors NUR77 and NOR1
Source: Chin Med. 2023 Sep 21;18:123. doi: 10.1186/s13020-023-00808-7 (PMC10512564; doi:10.1186/s13020-023-00808-7)
Supplement: Supplementary file 4 — Adtional file 4: Table S4. KEGG enrichment results of DEGs. [file 13020_2023_808_MOESM4_ESM.doc]

**Additional file 4:**

**Table S4**

KEGG enrichment results of DEGs.

| **ID** | **Description** | **GeneRatio** | **BgRatio** | **enrich_factor** | **pvalue** |
| --- | --- | --- | --- | --- | --- |
| ko00130 | Ubiquinone and other terpenoid-quinone biosynthesis | 9/591 | 27/7548 | 4.26 | 0.0001 |
| ko00051 | Fructose and mannose metabolism | 10/591 | 35/7548 | 3.65 | 0.0002 |
| ko04072 | Phospholipase D signaling pathway | 24/591 | 153/7548 | 2 | 0.0007 |
| ko04060 | Cytokine-cytokine receptor interaction | 36/591 | 282/7548 | 1.63 | 0.0022 |
| ko04620 | Toll-like receptor signaling pathway | 18/591 | 113/7548 | 2.03 | 0.0028 |
| ko04973 | Carbohydrate digestion and absorption | 10/591 | 48/7548 | 2.66 | 0.0034 |
| ko03008 | Ribosome biogenesis in eukaryotes | 14/591 | 85/7548 | 2.1 | 0.0058 |
| ko03010 | Ribosome | 21/591 | 150/7548 | 1.79 | 0.0063 |
| ko04064 | NF-kappa B signaling pathway | 17/591 | 114/7548 | 1.9 | 0.0071 |
