# Supplementary material for: Z-ligustilide preferentially caused mitochondrial dysfunction in AML HL-60 cells by activating nuclear receptors NUR77 and NOR1
Source: Chin Med. 2023 Sep 21;18:123. doi: 10.1186/s13020-023-00808-7 (PMC10512564; doi:10.1186/s13020-023-00808-7)
Supplement: Supplementary file 5 — Additional file 5: Table S5. A subset of mRNAs regluating glycolysis that are translationally up-regulated following Z-LIG treatment. [file 13020_2023_808_MOESM5_ESM.doc]

**Additional file 5:**

**Table S5**

A subset of mRNAs regluating glycolysis that are translationally up-regulated following Z-LIG treatment.

| **Gene Symbol** | **Gene ID** | **Gene Description** | **Fold Change** | |
| --- | --- | --- | --- | --- |
| **Glycolysis / Gluconeogenesis** | | | | |
| PCK2 | 5106 | phosphoenolpyruvate carboxykinase 2, mitochondrial | | 1.34 |
| ENO2 | 2026 | enolase 2 | | 3.36 |
| ACSS2 | 55902 | acyl-CoA synthetase short chain family member 2 | | 1.46 |
| ALDH1B1 | 219 | aldehyde dehydrogenase 1 family member B1 | | 1.95 |
| HK1 | 3098 | hexokinase 1 | | 1.56 |
| HK3 | 3101 | hexokinase 3 | | 3.93 |
| FBP1 | 2203 | fructose-bisphosphatase 1 | | 1.47 |
