# Supplementary material for: Z-ligustilide preferentially caused mitochondrial dysfunction in AML HL-60 cells by activating nuclear receptors NUR77 and NOR1
Source: Chin Med. 2023 Sep 21;18:123. doi: 10.1186/s13020-023-00808-7 (PMC10512564; doi:10.1186/s13020-023-00808-7)
Supplement: Supplementary file 6 — Additional file 6: Table S6. A subset of mRNAs encoding mitochondrial proteins that are translationally suppressed after Z-LIG treatment. [file 13020_2023_808_MOESM6_ESM.doc]

**Additional file 6:**

**Table S6**

A subset of mRNAs encoding mitochondrial proteins that are translationally suppressed after Z-LIG treatment.

| **Gene Symbol** | **Gene ID** | **Gene Description** | **Fold Change** | |
| --- | --- | --- | --- | --- |
| **Translation elongation factor** | | | | |
| TSFM | 10102 | Ts translation elongation factor, mitochondrial | | -1.66 |
| **Oxidative Phosphorylation Complex Ⅰ** | | | | |
| NDUFAF6 | 137682 | NADH:ubiquinone oxidoreductase complex assembly factor 6 | | -1.52 |
| NDUFAF4 | 29078 | NADH:ubiquinone oxidoreductase complex assembly factor 4 | | -1.95 |
| NDUFAF2 | 91942 | NADH:ubiquinone oxidoreductase complex assembly factor 2 | | -1.27 |
| **Oxidative Phosphorylation Complex Ⅲ** | | | | |
| UQCC3 | 790955 | ubiquinol-cytochrome c reductase complex assembly factor 3 | | -1.22 |
| **Oxidative Phosphorylation Complex Ⅴ** | | | | |
| ATP1A3 | 478 | ATPase Na+/K+ transporting subunit alpha 3 | | -2.95 |
| ATP5G1 | 516 | ATP synthase membrane subunit c locus 1 | | -1.33 |
| **Mitochondrial tRNA modifucation** | | | | |
| GTPBP3 | 84705 | GTP binding protein 3, mitochondrial | | -1.54 |
| **Maturation of Primary Transcription** | | | | |
| PNPT1 | 87178 | polyribonucleotide nucleotidyltransferase 1 | | -1.28 |
| TRMT1 | 55621 | tRNA methyltransferase 1 | | -1.30 |
| TRMT10C | 54931 | tRNA methyltransferase 10C, mitochondrial RNase P subunit | | -1.46 |
| TRMT61A | 115708 | tRNA methyltransferase 61A | | -1.30 |
| MRM1 | 79922 | mitochondrial rRNA methyltransferase 1 | | -2.33 |
| SLIRP | 81892 | SRA stem-loop interacting RNA binding protein | | -1.49 |
| **Mitochondrial Translation** | | | | |
| MRPL12 | 6182 | mitochondrial ribosomal protein L12 | | -1.61 |
| MRPS34 | 65993 | mitochondrial ribosomal protein S34 | | -1.47 |
| MRPS12 | 6183 | mitochondrial ribosomal protein S12 | | -1.35 |
| MRPL4 | 51073 | mitochondrial ribosomal protein L4 | | -1.82 |
| MRPL15 | 29088 | mitochondrial ribosomal protein L15 | | -1.40 |
| MRPL24 | 79590 | mitochondrial ribosomal protein L24 | | -1.77 |
| MRPL1 | 65008 | mitochondrial ribosomal protein L1 | | -1.81 |
| MRPS21 | 54460 | mitochondrial ribosomal protein S21 | | -1.23 |
| MRPS17 | 51373 | mitochondrial ribosomal protein S17 | | -1.86 |
| MRPL54 | 116541 | mitochondrial ribosomal protein L54 | | -1.27 |
| MRPL47 | 57129 | mitochondrial ribosomal protein L47 | | -1.48 |
| MRPL23 | 6150 | mitochondrial ribosomal protein L23 | | -1.27 |
| MRPS25 | 64432 | mitochondrial ribosomal protein S25 | | -1.55 |
