# Supplementary material for: Z-ligustilide preferentially caused mitochondrial dysfunction in AML HL-60 cells by activating nuclear receptors NUR77 and NOR1
Source: Chin Med. 2023 Sep 21;18:123. doi: 10.1186/s13020-023-00808-7 (PMC10512564; doi:10.1186/s13020-023-00808-7)
Supplement: Supplementary file 7 — Additional file 7: Fig. S1. Potential target genes of Z-LIG based on RNA-seq. [file 13020_2023_808_MOESM7_ESM.doc]

**Additional file 7:**

**
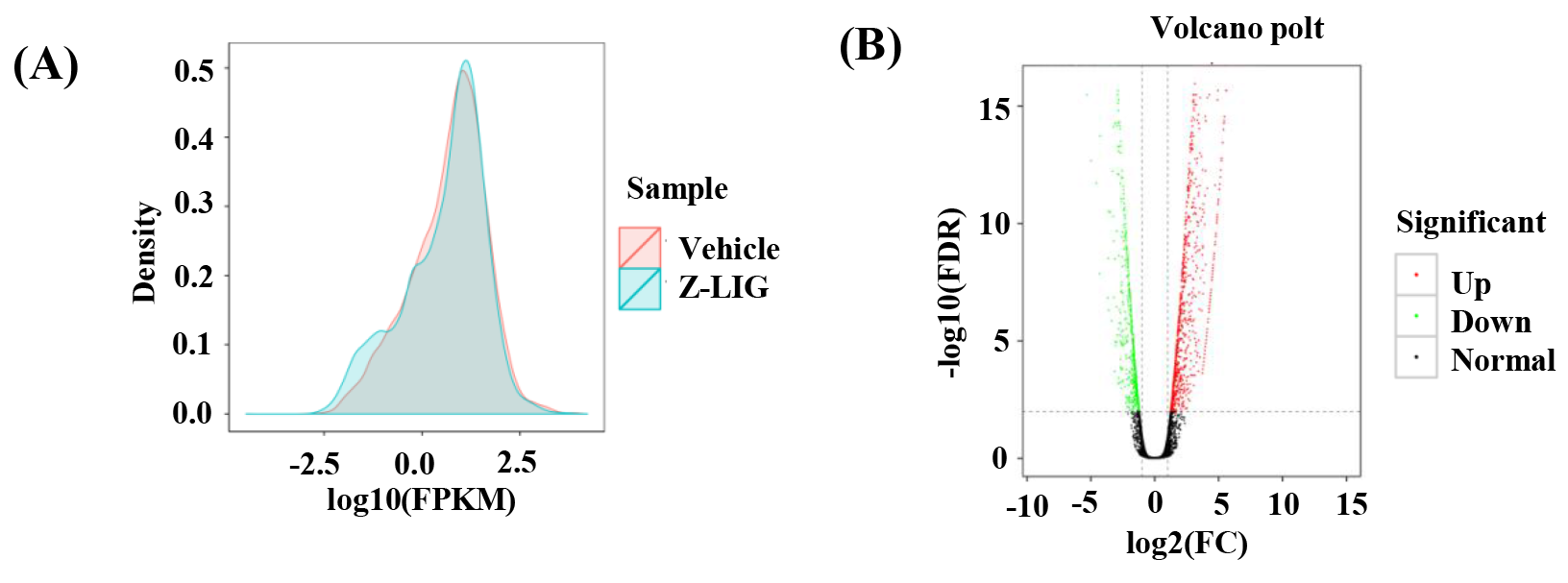
**

**Fig. S1** Potential target genes of Z-LIG based on RNA-seq. RNA-seq was performed in HL-60 cells after the treatment with vehicle or Z-LIG (25  μM for 24  h). (A) Comparison chart of FPKM density distribution of sample. (B) Volcano map of differential expressed genes.
