# Supplementary material for: Z-ligustilide preferentially caused mitochondrial dysfunction in AML HL-60 cells by activating nuclear receptors NUR77 and NOR1
Source: Chin Med. 2023 Sep 21;18:123. doi: 10.1186/s13020-023-00808-7 (PMC10512564; doi:10.1186/s13020-023-00808-7)
Supplement: Supplementary file 8 — Additional file 8: Fig. S2. Z-LIG induced mitochondrial division in HL-60 cells. [file 13020_2023_808_MOESM8_ESM.doc]

**Additional file 8:**

**
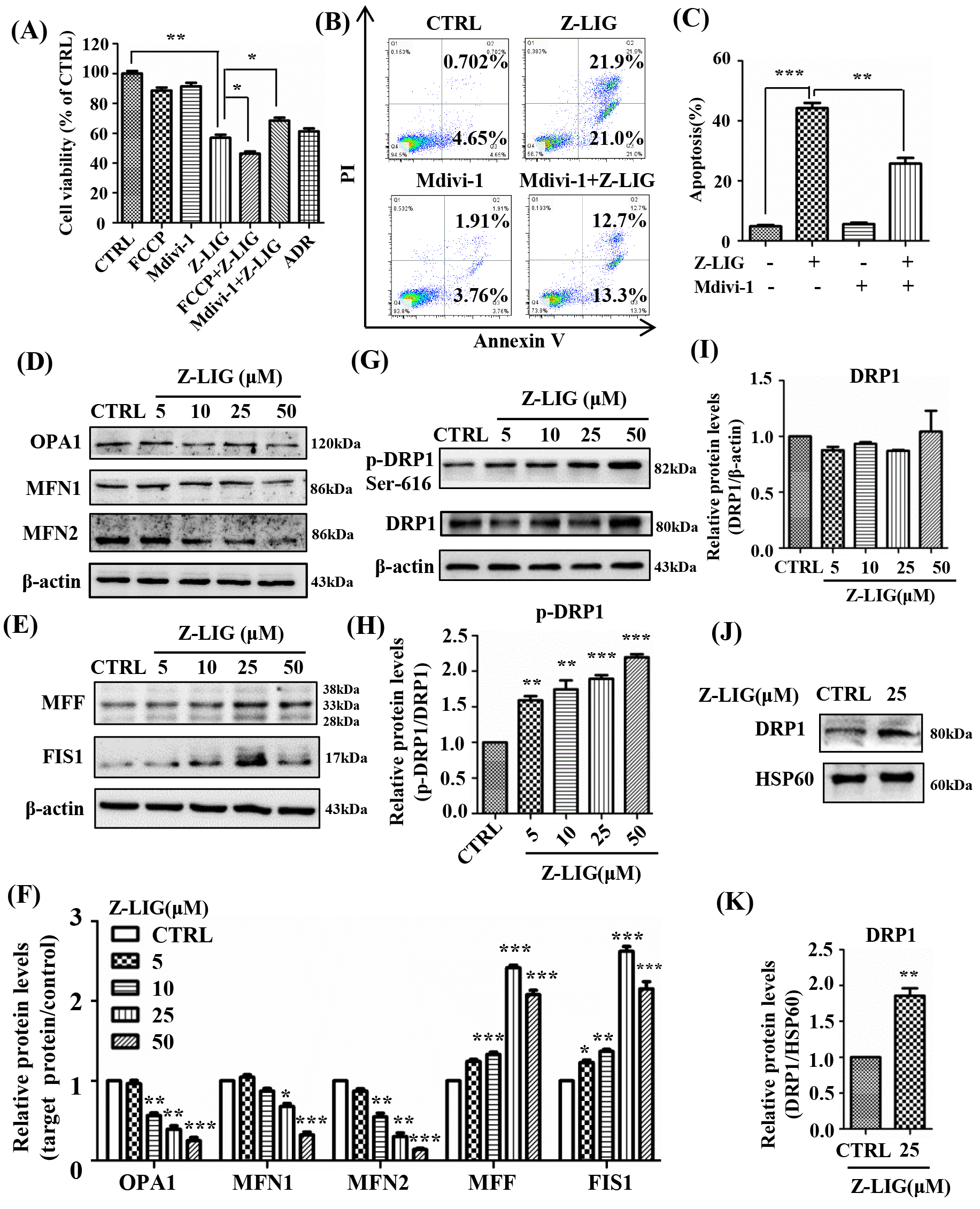
**

**Fig. S2** Z-LIG induced mitochondrial division in HL-60 cells. (A-C) HL-60 cells were pretreated with Mdivi-1 (2.5 μM) or FCCP (2.0 μM) or vehicle for 1 h, and then treated with Z-LIG (25 μM) or ADR (32 nM) or vehicle. Cell viability was assessed by SRB after 72 h treatment and apoptotic cells were determined by flow cytometry after 48 h treatment. (D-I) HL-60 cells were treated with Z-LIG for 48 h and then cell lysates were subjected to western blotting for the detection of the proteins as indicated. F was densitometric analyses of the western blots shown in D and E. H and I were densitometric analyses of the western blots shown in G. (J) Mitochondrial fractions were subjected to western blotting for the detection of the proteins as indicated. (K) Densitometric analyses of the western blots in J. Values are presented as the means ± SD (n = 3 per group). **P* < 0.05, ** *P* < 0.01, and *** *P* < 0.001 vs CTRL or indicated group.
